# The Role of Androgen Receptor in Cross Talk Between Stromal Cells and Prostate Cancer Epithelial Cells

**DOI:** 10.3389/fcell.2021.729498

**Published:** 2021-10-06

**Authors:** Qianyao Tang, Bo Cheng, Rongyang Dai, Ronghao Wang

**Affiliations:** ^1^Department of Biochemistry and Molecular Biology, School of Basic Medical Sciences, Southwest Medical University, Luzhou, China; ^2^Department of Urology, The Affiliated Hospital of Southwest Medical University, Luzhou, China

**Keywords:** PCa, AR, microenvironment, stroma, androgen deprivation therapy (ADT)

## Abstract

Prostate cancer (PCa) lists as the second most lethal cancer for men in western countries, and androgen receptor (AR) plays a central role in its initiation and progression, which prompts the development of androgen deprivation therapy (ADT) as the standard treatment. Prostate tumor microenvironment, consisting of stromal cells and extracellular matrix (ECM), has dynamic interactions with PCa epithelial cells and affects their growth and invasiveness. Studies have shown that both genomic and non-genomic AR signaling pathways are involved in the biological regulation of PCa epithelial cells. In addition, AR signaling in prostate stroma is also involved in PCa carcinogenesis and progression. Loss of AR in PCa stroma is clinically observed as PCa progresses to advanced stage. Especially, downregulation of AR in stromal fibroblasts dysregulates the expression levels of ECM proteins, thus creating a suitable environment for PCa cells to metastasize. Importantly, ADT treatment enhances this reciprocal interaction and predisposes stromal cells to promote cell invasion of PCa cells. During this process, AR in PCa epithelium actively responds to various stimuli derived from the surrounding stromal cells and undergoes enhanced degradation while elevating the expression of certain genes such as MMP9 responsible for cell invasion. AR reduction in epithelial cells also accelerates these cells to differentiate into cancer stem-like cells and neuroendocrine cells, which are AR-negative PCa cells and inherently resistant to ADT treatments. Overall, understanding of the cross talk between tumor microenvironment and PCa at the molecular level may assist the development of novel therapeutic strategies against this disease. This review will provide a snapshot of AR’s action when the interaction of stromal cells and PCa cells occurs.

## Introduction

Prostate cancer (PCa) is a malignant growth of prostate epithelial cells, and it continuously causes severe mortality among men. According to a survey in 2020, an estimated 190,000 new cases of PCa are diagnosed and 33,000 associated deaths are reported worldwide (Siegel et al., 2020), suggesting an urgent need to identify effective therapeutic strategies against this disease. Given the fact that androgen receptor (AR) plays central roles in PCa carcinogenesis (Heinlein and Chang, 2004; Lonergan and Tindall, 2011), androgen deprivation therapy (ADT) is the mainstream treatment for PCa, which is effective for 2–3 years before PCa progresses to castration-resistant PCa (CRPC; Ruizeveld de Winter et al., 1994; Henshall et al., 2001; Scher et al., 2004). The reactivation of AR in CRPC, owing to gene amplification (Koivisto et al., 1997), gene mutation (Tilley et al., 1996), or the production of constitutively active AR variants (Nakazawa et al., 2014), makes it still a promising therapeutic target.

**FIGURE 1:**
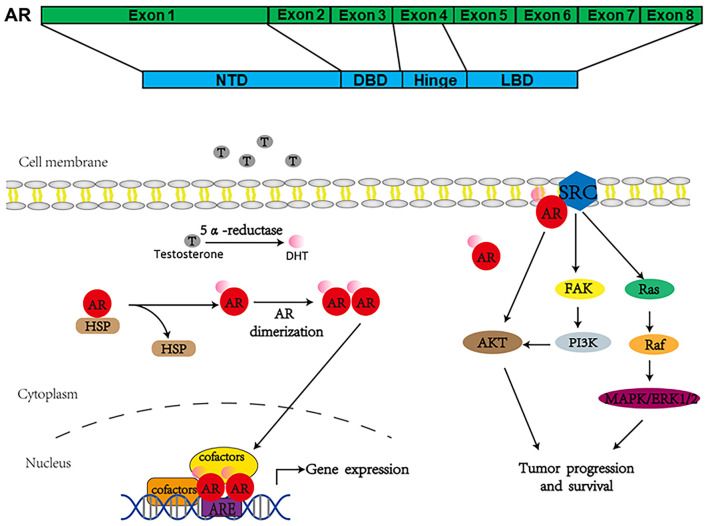
Androgen receptor’s structure and how it works to regulate gene expression.

**FIGURE 2:**
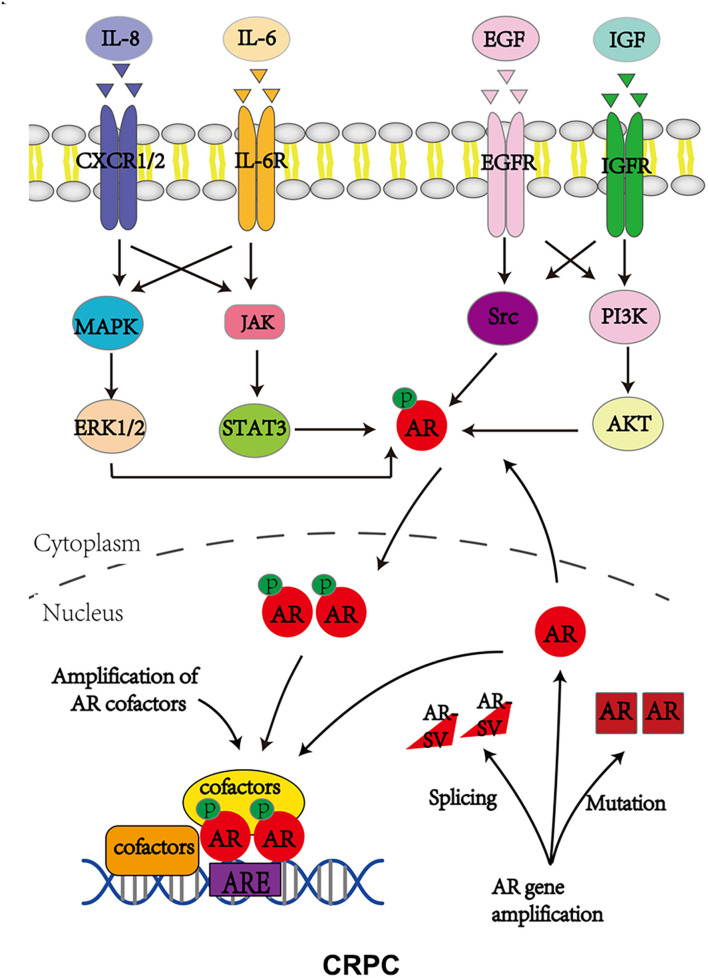
Androgen receptor utilizes various ways to bypass AR inhibition to support CRPC growth. P stands for phosphorylation.

Androgen receptor, also called NR3C4 (nuclear receptor subfamily 3, group C, member 4), is one member of the steroid and nuclear receptor superfamily and is encoded by the AR gene located at Xq11-12 (Tan et al., 2015). AR mainly contains four functional domains, as indicated in Figure 1: the amino-terminal domain (NTD; exon 1), DNA-binding domain (DBD; exons 2–3), hinge region (exon 4), and ligand-binding domain (LBD; exons 4–8). As a hormone-inducible transcription factor, AR is ubiquitously expressed in multiple tissues and is involved in various physiological and pathological events by controlling gene expression (Waghray et al., 2001; DePrimo et al., 2002). Genomic AR signaling in PCa is well studied: AR disassociates from its cytoplasmic chaperones such as HSP70 and translocates to the nucleus upon androgenic hormone binding, working as either homodimer or heterodimer to regulate its downstream genes such as *PSA*, *FKBP5*, and *TMPRSS2*, in order to provide survival signals for PCa growth (Figure 1; Smith and Toft, 2008). Of note, activation of non-genomic AR signaling is also critical to PCa development. An early study demonstrated that AR in both prostatic smooth muscle cells and prostatic epithelial cells rapidly responded to androgen stimulation (Peterziel et al., 1999) and interacted with non-receptor tyrosine kinase Src (Migliaccio et al., 2000; Castoria et al., 2017). This interaction impaired the inhibitory intramolecular binding of Src and allowed it in its active form to activate PI3K and MAPK signaling pathways, which promoted cell proliferation and cell invasion of PCa (Cai H. et al., 2011; Castoria et al., 2017). Although castration leads to a remarkable decline of AR activity, AR utilizes various novel ways to escape androgen ablation and continues to support PCa growth in castration-resistant stage (Figure 2). For instance, upregulated EGF and IGF in CRPC could activate AR transactivation. Mechanistically, Src responded to EGF stimulation and phosphorylated AR at the residue of tyrosine 534, leading to AR activation (Migliaccio et al., 2005; Liu et al., 2010). IGF-stimulated AKT and ERK1/2 signaling pathways were directly responsible for AR activation (Wu et al., 2006). In addition, elevated cytokines in CRPC such as IL-6 or IL-8 also had the capacity to activate AR signaling *via* MAPK and JAK/STAT3 signaling pathways (Chen et al., 2000; Seaton et al., 2008). Studies also revealed that AR mutation including aberrant AR splicing and AR gene amplification were frequently (10–30%) observed in CRPC patients (Visakorpi et al., 1995; Tilley et al., 1996; Koivisto et al., 1997), which allowed it to be active even under castrated conditions, effectively conferring androgen independence to AR.

Prostate cancer is a malignant mass infiltrated with endothelial cells, bone marrow stem cells, fibroblasts, and immune cells (Sejda et al., 2020; Thienger and Rubin, 2021). Evidence suggested that the infiltration of these cells increased as PCa progressed to the CRPC stage. These stromal cells provide cytokines and growth factors to drive PCa progression. Clinical evidence indicated that stromal AR was a protective factor for PCa patients as its expression was inversely related to poor PCa outcomes including tumor stage, metastasis, cancer relapse, and cancer-related death (Olapade-Olaopa et al., 1999; Henshall et al., 2001; Ricciardelli et al., 2005; Li et al., 2008; Wikstrom et al., 2009; Leach et al., 2015). In contrast, AR in PCa epithelium acted as a tumor-promoting factor to control PCa development. Therefore, comprehensive knowledge of AR’s role in the cross talk between PCa and its surrounding stromal component is necessary for scientists to better understand this disease.

## Androgen Receptor Inhibition as a Standardized Treatment for Prostate Cancer

Androgen receptor inhibition is the major way to treat PCa, and AR-targeted therapies are listed in Table 1. ADT has been the mainstay treatment for PCa patients in past decades, which includes surgical and chemical castration. Surgical castration refers to one operation in which the entire testes is removed to ablate androgen synthesis, while chemical castration utilizes various types of compounds to antagonize AR signaling pathway in PCa. Leuprorelin and triptorelin are clinically used as LHRH agonists to fulfill androgen deprivation (Crawford and Hou, 2009; Lepor and Shore, 2012). Mechanistically, LHRH agonist treatment leads to LHRH downregulation and eventually inhibits pituitary production of follicle-stimulating hormone (FSH) and luteinizing hormone (LH), two important hormones responsible for androgen synthesis in testes, while LHRH antagonists such as degarelix and relugolix interact with LHRH receptor and impair the physiological binding of LHRH and its receptor, reducing androgen synthesis. However, LHRH agonists and antagonists only block testes from producing androgens. Evidence show that 5–10% of androgens are generated from adrenal glands and they are sufficient to drive AR signaling (Montgomery et al., 2008), which facilitates the development of other compounds in order to reduce the synthesis of adrenal androgens. CYP17 is a key enzyme required for the biosynthesis of adrenal androgens so that its specific inhibitors such as abiraterone, TAK-700, and TOK-001 have been developed to treat CRPC patients who are resistant to LHRH agonists and antagonists (Schweizer and Antonarakis, 2012).

**TABLE 1:** Androgen receptor targeted therapies in prostate cancer.

Established therapies	AR based drugs	Type of PCa	Mechanistic action
LHRH agonists	Leuprolide; Leuprolide mesylate; Triptorelin; Goserelin; Histrelin	Localized	Suppress LHRH expression and reduce the production of FSH and LH. Impair androgen synthesis in testes
LHRH antagonists	Degarelix; Relugolix	Localized; Advanced	Compete LHRH to bind its receptor and prevent its biological function
CYP17 inhibitors	Abiraterone; TAK-700 (Orteronel); Ketoconazole;	mCRPC	Inhibition of biosynthesis of adrenal androgens by suppressing CYP17 activity
	TOK-001 (Galeterone)	CRPC	
Antiandrogen	Bicalutamide; Nilutamide; Flutamide	Localized; Advanced	First generation antiandrogen, compete androgen to interact with AR
	Enzalutamide	mCRPC	Second generation antiandrogen, compete androgen to interact with AR, block AR nuclear translocation, inhibit AR DNA binding and the recruitment of AR cofactors
	Apalutamide (ARN-509)	mCSPC	
	Darolutamide	nmCRPC	

*CRPC stands for castration resistant prostate cancer; mCRPC means metastatic castration resistant prostate cancer; nmCRPC means non-metastatic castration resistant prostate cancer; mCSPC stands for metastatic castration sensitive prostate cancer.*

Of note, the castration level of androgen still has the ability to activate AR signaling and to support CRPC growth. To reach a deep AR inhibition, anti-androgens are developed to treat metastatic CRPC patients. They directly inhibit the binding of androgen to AR and to prevent AR nuclear translocation and the subsequent transactivation. However, first-generation anti-androgens such as bicalutamide cannot completely block AR activity due to their relatively low affinity to AR. This leads to the emergence of second-generation anti-androgens in the standard treatment of metastatic CRPC patients. Enzalutamide is one of these second-generation anti-androgens and significantly improves patients’ survival (Scher et al., 2010; Roumiguie et al., 2021). Overall, AR inhibition is the main strategy to prevent PCa development.

## Androgen Receptor Signaling in Cross Talk Between Endothelial Cells and Prostate Cancer

Endothelial cells are key builders of vascular structure, and their proliferation, migration, and remodeling are tightly associated with angiogenesis (Dudley, 2012). Early reports demonstrated that initial ADT treatment led to the apoptosis of endothelial cells and decreased the number of microvascules (Shabsigh et al., 1998). Very intriguingly, microvascules showed regrowth and the number of endothelial cells evidently increased as PCa progressed to the CRPC stage (Godoy et al., 2011; Tomic et al., 2012). This regrowth of microvascules was accompanied by PCa metastasis. Indeed, *in vitro* evidence supported the notion that endothelial cells could increase PCa cell migration and invasion. By coculturing HUVECs (human umbilical vein endothelial cells) with PCa cells, Chang et al. found that HUVECs had the capacity to enhance cell migration and cell invasion of CWR22Rv1 and C4-2 cells (Wang et al., 2013). According to their data, PCa cells exposed to HUVECs expressed lower levels of AR than non-exposed controls. AR seemingly regulated TGFβ and MMP9 in a negative manner, as evidenced by the enhanced expression levels of TGFβ and MMP9 when AR was depleted by siRNAs. They also identified that IL-6, a cytokine secreted by HUVECs, was sufficient to drive AR downregulation. Consistent with this report, HUVEC-enhanced cell invasion of PCa cells *via* downregulating the AR level was also observed by Jiang et al. AR reduction was promoted by the interaction of CCL5 on endothelial cells with its receptor CCR5 on PCa cells, triggering mitochondria-mediated autophagy, one mechanism responsible for cell invasion of PCa cells (Zhao et al., 2018). Interestingly, another study documented that MMP9, together with PIP5K1α, could interact with AR to promote its transcriptional activity on the downstream target cyclin A1, leading to enhanced growth of metastatic CRPC tumor (Larsson et al., 2020). However, as a transcription factor, how AR regulates TGFβ, MMP9, and autophagy in the presence of HUVECs is still largely unknown and requires further exploration. In addition, it remains to be clarified how IL-6 and CCL5/CCR5 signals at a molecular level reduce AR expression.

On the other hand, anti-androgen-resistant PCa tends to express a higher level of endothelial nitric oxide synthase (eNOS) compared to hormone naive PCa (Yu et al., 2013), suggesting that chronic inhibition of AR signaling causes an upregulation of eNOS, a critical molecule involved in supporting endothelial cell survival and proliferation. Together, all these data pinpoint a positive feedback loop between PCa cells and endothelial cells during PCa development.

How does AR function in endothelial cells? Literature revealed that androgen could promote cell proliferation of HUVECs by activating the AR/VEGF/Cyclin A signaling axis (Cai J. et al., 2011; Torres-Estay et al., 2017; Eisermann et al., 2013). However, another study contradictorily showed that AR activation triggered TNF-α-induced cell apoptosis of EA.hy926 endothelial cells (Ling et al., 2002). One possible explanation to this is that different endothelial cell lines have distinct responses to androgen treatment. Therefore, the exact role of AR in endothelial cells during PCa progression is still open to investigation.

## Interaction Between Bone Marrow Mesenchymal Stem Cells and Androgen Receptor Signaling in Prostate Cancer

An observation of the migration of mesenchymal stem cells (BM-MSCs) into PCa stroma implies their possible participation in the regulation of PCa development (Placencio et al., 2010; Brennen et al., 2017). As multipotent stromal cells, BM-MSCs bear the ability to differentiate into various cell types (Pittenger et al., 1999; Mendez-Ferrer et al., 2010) including fibroblasts, adipocytes, and smooth muscle cells in PCa stroma. Moreover, the recruited BM-MSCs promoted cell invasion and stemness differentiation of PCa cells, ultimately allowing PCa cells to survive in an androgen-independent manner (Chang et al., 2014; Luo et al., 2014, 2015). Mechanistic dissection illustrated that BM-MSCs could secrete CCL5 and IL-1β, which independently acted to reduce the AR level. According to the literature, secreted CCL5 from BM-MSCs led to decreased levels of prolyl hydroxylase (PHD) 1 and 4, which prevented the hydroxylation of HIF2α and caused it to become more stable (Luo et al., 2015). Immunoprecipitation assay also confirmed that CCL5-induced HIF2α enhanced HSP70-AR interaction, which inhibited AR nuclear translocation and the subsequent transactivation. Another study also verified that conditioned medium from BM-MSCs could decrease the AR protein level, which was mediated by the cytokine IL-1β(Chang et al., 2014). However, the detailed mechanism by which IL-1βdecreased AR was not clearly explored. These studies also proved that the reduction of AR was a central driving force to promote PCa cell invasion. Further explorations revealed that AR downregulation by BM-MSCs increased the stemness of PCa, owing to the upregulation of several CSC (cancer stem cells) related markers such as CXCR4, ZEB1, and Snail1 (Luo et al., 2014). Given the fact that prostatic CSCs are more malignant than parental PCa cells, it is understandable that BM-MSCs promote PCa invasion and metastasis.

Of note, a lack of evidence showing that AR in BM-MSCs exerts its function to regulate PCa survival and invasion may prompt scientists to explore this direction.

## Interaction Between Androgen Receptor Signaling in Cancer-Associated Fibroblasts and Prostate Cancer

Cancer-associated fibroblasts (CAFs), one type of activated fibroblasts, typically express high levels of αSMA (α-smooth muscle actin) and fibroblast activation protein (FAP; Sahai et al., 2020). Studies indicate that CAFs have a malignant property by affecting their surrounding compartments including cancerous cells and other stromal cells (Taylor et al., 2012). Compared to normal fibroblasts, CAFs have distinct genome-wide DNA methylation signatures at enhancers and promoters, causing aberrant expression of cancer-related genes to impact cancer fate (Pidsley et al., 2018). Abundant studies show that CAFs exist in the primary site of localized or metastatic PCa microenvironment and promote its initiation and progression. By comparing immortalized CAFs and normal prostate fibroblasts, researchers noticed that CAFs had stronger ability to promote malignant transformation of BPH-1 cells *in vitro* and *in vivo* (Yu et al., 2017), suggesting that CAF was a central driving source for PCa initiation. In this process, AR in stromal fibroblasts but not in epithelium is necessary for prostatic epithelia malignant transformation (Ricke et al., 2012). According to this study, loss of stromal AR did not impair prostate homeostasis but decreased the expression levels of several stroma fibroblast-derived growth factors such as FGF-2 and FGF-10, impeding PCa carcinogenesis (Ricke et al., 2012). Moreover, studies also indicated that CAFs had better capacity to increase cell proliferation and invasion of LNCaP cells *in vitro* (Yu et al., 2017), compared to normal prostate fibroblasts. The LNCaP xenografted mouse model also strengthened the tumor-promoting role of CAFs in PCa carcinogenesis (Yu et al., 2017). Consistent with this report, another study also found that CAFs significantly enhanced PCa growth and distant metastases when compared to normal prostate fibroblasts (Linxweiler et al., 2020). Why are CAFs so effective in promoting PCa development? Focus cytokine array showed that CAFs secreted more growth factors such as EGF, FGF, HGF, TGFβ and VEGF, than normal prostate fibroblasts, providing survival advantage to PCa cells (Yu et al., 2017).

How does AR contribute to fibroblast proliferation and migration? Results from mouse embryo fibroblasts NIH3T3 and fibrosarcoma HT1080 revealed that androgen stimulation (10 nM) could suppress proliferation while increasing migration of these two cell lines (Castoria et al., 2014). Mechanistic dissection unfolded that the androgen-stimulated AR/FlnA complex led to the activation of Rac1 as well as its downstream effector DYRK 1B, which phosphorylated p27 at Ser 10. The phosphorylated p27 at Ser 10 was stabilized and attributable to cell cycle arrest at G0/G1 of fibroblasts (Castoria et al., 2014). Meanwhile, the AR/FlnA complex also triggered the activation of focal adhesion kinase FAK, Rac1, and paxillin *via* recruiting integrin beta 1, promoting cell migration of NIH3T3, HT1080 cells, and PCa-derived fibroblasts (Castoria et al., 2011; Di Donato et al., 2021). These findings suggest the therapeutic value of targeting the AR/FlnA complex to suppress the malignant activity of CAFs. Indeed, knockdown of FlnA with siRNAs or inhibition of AR by enzalutamide could abolish the migration and invasiveness of CAFs induced by androgen treatment (Di Donato et al., 2021). Similarly, disruption of the AR/FlnA complex by drug-like compound Rh-2025u-stapled peptide, which was developed from the AR amino acid sequence required for the interaction with FlnA (Loy et al., 2003; Castoria et al., 2011), abolished the androgen-induced migration and invasiveness of CAFs, consequently leading to less recruitment of CAFs to PCa and suppressing PCa-CAF organoid growth (Di Donato et al., 2021). How does AR in CAFs function to influence PCa growth and invasion? Several studies have demonstrated that the reduced expression level of AR in CAFs was a prognostic indicator of PCa progression, suggesting that AR in CAFs serves as a negative regulator to determine PCa development. A study showed that AR regulated extracellular matrix (ECM) components and maintained its inhibition on PCa cell invasion. Loss of AR in fibroblasts led to the downregulation of adhesive proteins such as FBXO32 and FBN1 as well as the upregulation of ECM-degrading enzyme MMP1, establishing an environment permissive for PCa cells to invade (Leach et al., 2015). By using the *in vitro* coculture system, Smith and colleagues found that immortalized human prostate myofibroblast cell line PShTert with AR activation could retard the cell growth of various PCa cells including PC3, Du145, C4-2B, and LNCaP cells, highlighting the protective role of AR in CAFs during PCa development (Palethorpe et al., 2018). Additionally, another report further proved that loss of AR in CAFs promoted cell migration of PCa cells (Cioni et al., 2018). As described in this report, AR negatively regulated the expressions of CCL2 and CXCL8 at the transcriptional level. Therefore, loss of AR released its inhibitory regulation on these two cytokines, which were secreted and promoted PCa cell migration in a paracrine manner. Another document strengthened this conclusion by pinpointing that loss of AR in CAFs led to the enhanced expression levels of interferon gamma (IFN-γ) and macrophage colony-stimulating factor (M-CSF), which acted on PCa cells and educated them to differentiate into cancer stem-like cells (Liao et al., 2017) characterized with more invasive properties. Contradictorily, the group of Chang illustrated that myofibroblast stromal cell line WPMY-1 with AR siRNAs could suppress the cell invasion of PCa cells in the coculture system (Niu et al., 2008), and the group of Gross described that reduction of AR in CAFs with antisense oligonucleotide (ASO) dramatically suppressed CAF-promoted PCa growth *in vitro* and *in vivo* (Liao et al., 2017). It can be argued that the group of Chang reached the conclusion on the basis of an artificial coculture system. In the study by Gross, ASOs were utilized to reduce the AR protein level, while the previous study applied anti-androgen RD162 for AR inhibition. It is possible that the physiological effects of anti-androgen and ASO on CAFs are different so that opposite conclusions are generated. Besides, Gross et al. utilized mouse CAFs as a source to study their contribution to the cell growth of human PCa cells, which was not an optimal *in vitro* model.

Although AR signaling is very important for PCa growth and metastasis, few studies examined AR status in PCa epithelial cells that interprets the signal from CAFs to influence PCa epithelial cells when these two types of cells are communicating with each other, which should be explored with future efforts.

## Androgen Receptor Signaling in Cross Talks Between Immune Cells and Prostate Cancer

Infiltration of immune cells into PCa is frequently observed, which is further increased upon ADT treatment, indicating that immune cells are actively involved in PCa progression. Macrophages, T lymphocytes, NK cells, and mast cells are the most important components of immune cells, and their cross talk with AR signaling in PCa will be summarized as follows.

## Androgen Receptor Signaling in Cross Talk Between CD4+T Cells and Prostate Cancer

T cells consist of CD8 + cytotoxic T cells and CD4 + helper T cells, playing a central role in the adaptive immune response. Kwon et al. demonstrated that androgen ablative therapy (ADT) could increase the infiltration of CD4 + T cells but not CD8 + T cells into the PCa (Mercader et al., 2001). In agreement with this observation, Underwood et al. also found that the infiltration of CD4 + T cells was strongly associated with poor outcome in PCa patients (McArdle et al., 2004). Collectively, all these findings suggest that CD4 + T cells may participate in PCa progression. Consistently, another study documented that androgen (testosterone) could inhibit the differentiation of CD4 + T cells (Kissick et al., 2014). CD4 + T cells isolated from castrated mice exhibited reduced STAT4 phosphorylation and IFN-γ production upon testosterone treatment. Testosterone activated AR to transcriptionally upregulate the expression level of Ptpn1 (protein tyrosine phosphatase non-receptor type 1), which in turn served as a negative regulator of STAT4 signaling (Kissick et al., 2014). Therefore, castration not only increased the infiltration of CD4 + T cells but also promoted their differentiation. Collectively, these data suggest that CD4 + T cells may play a tumor-promoting role in PCa development. Indeed, through an *in vitro* cell model, Chang et al. found that PCa cells had a better capacity to recruit CD4 + HH and Molt-3 T cells compared to normal prostate epithelial RWPE-1 cells (Hu et al., 2015). Mechanistically, FGF11 secreted from HH and Molt-3 T cells led to the reduction of AR in PCa cells by enhancing miR-541-mediated AR mRNA degradation (Hu et al., 2015). AR downregulation could elevate the MMP9 expression level, promoting PCa invasion and metastasis. Again, detailed mechanisms underlying AR regulation of MMP9 were not investigated in this study. In addition, it was unlikely that FGF11 directly increased the expression level of miR-541 to reduce the AR level. PCa cells must undergo multiple signaling events upon FGF11 treatment to complete miR-541 induction.

Although ADT with enzalutamide treatment initially increased the infiltration of CD4 + helper cells, it sensitized PCa cells to CD8 + T cell killing by downregulating the expression of antiapoptotic gene NAIP (neuronal apoptosis inhibitory protein) (Ardiani et al., 2014). NAIP played a critical role in conferring the killing ability of CD8 + cytotoxic cells toward PCa cells. In enzalutamide-resistant PCa, AR signaling was reactivated in various ways so that AR drove NAIP expression, decreasing the sensitivity of PCa cells to CD8 + cytotoxic cells (Ardiani et al., 2014). Therefore, a combined therapy using T cell immunotherapy with anti-androgen therapy becomes ideal for PCa management (Sanchez et al., 2013).

## Androgen Receptor Signaling in Cross Talk Between Macrophages and Prostate Cancer

As the most predominant immune cells within the PCa microenvironment, macrophages play a central role in PCa development including cell survival, cell invasion, angiogenesis, lineage plasticity, and anti-androgen resistance. Macrophages can be classified into pro-inflammatory/anti-tumoral M1 type and anti-inflammatory/pro-tumoral M2 type. Typically, M2 macrophages, featured with high IL-10 production, increasingly infiltrate PCa, and this infiltration is strongly correlated with PCa aggressiveness (Comito et al., 2014). Studies demonstrated that PCa could promote differentiation and polarization of macrophages. PCa-derived IL-6, SDF1, and antimicrobial peptide LL-37 (leucin leucin 37) could promote M1–M2 differentiation/polarization (Comito et al., 2014; Cha et al., 2016), which in turn increased PCa invasiveness. Indeed, depletion of M2 macrophages remarkably inhibited tumor progression in various mouse tumor models (Sica et al., 2008), including PCa. Previous studies revealed that there was much more macrophage infiltration in PCa compared to matched normal tissues, monitored by the specific macrophage marker CD68 (Zhu et al., 2006; Fang et al., 2013), suggesting that macrophages may play a role in PCa tumorigenesis. In an experimental setting mimicking *in vivo* cell–cell interaction, Fang et al. (2013) applied the coculture system using immortalized prostate epithelial cells (RWPE-1) and macrophages (THP-1) to induce prostate tumorigenesis. The results demonstrated that RWPE-1 cells cocultured with THP-1 cells could well differentiate into prostate spheres and had better ability to develop tumor in xenografted mouse models, and strengthening macrophage infiltration can promote PCa tumorigenesis.

Castration further increased the infiltration of M2 macrophages into PCa (Lin et al., 2013; Yuri et al., 2020), and this recruitment of macrophages by ADT may be attributable to CCL2 production from PCa cells (Tsai et al., 2018). One publication illustrated that a transcriptional repressor of CCL2, SPDEF (SAM pointed domain-containing ETS transcription factor), was positively regulated by AR. ADT inactivated AR transcriptional activity to reduce the transcription of SPDEF, which in turn promoted the expression of CCL2. CCL2 bound its receptor CCR2 on macrophages in a paracrine manner and enhanced their recruitment to PCa cells (Tsai et al., 2018). Furthermore, the infiltrated macrophages would enhance PCa invasion/metastasis *via* downregulating AR and activating STAT3 signaling. Chang et al. found that coculture of THP-1 with PCa cells led to AR reduction in PCa cells (Izumi et al., 2013). They also identified that PIAS3 was an AR-inducible gene, which was transcriptionally decreased in the presence of THP-1 cells. Without the negative regulator PIAS3, STAT3 signaling was activated and drove PCa invasion/metastasis. Nevertheless, how AR is reduced when PCa cells receive the signals from macrophages remains unknown and requires additional investigation.

Androgen receptor seemingly acts as a tumor-suppressing factor in PCa-associated macrophages. According to Izumi et al. (2013), AR knockdown in THP-1 cells promoted the expression of CCL2, which bound its receptor CCR2 on PCa cells and activated STAT3-mediated EMT (epithelial mesenchymal transition) and cell invasion/metastasis. However, studies also showed that androgen had the capacity to promote M2 macrophage polarization (Becerra-Diaz et al., 2018; Larsson et al., 2020), supporting the tumor-promoting role of AR signaling in PCa-associated macrophages. Indeed, conditioned medium from PMA-activated THP-1 cells with R1881 stimulation could promote cell migration and cell invasion of CWR-R1 PCa cells without affecting their proliferating ability *in vitro* (Cioni et al., 2020). By applying ChIP-seq and Ingenuity Pathway Analysis, they identified that TREM-1 signaling was remarkably enriched in R1881-treated THP-1 cells compared to vehicle-treated ones, and AR physically bound to the proximal or distal upstream region of various genes related to TREM-1 signaling, implying that AR transcriptionally regulates the expression levels of these genes to control TREM-1 signaling. Further explorations revealed that several chemokines such as CCL2, CCL7, CCL13, and CXCL8 secreted by R1881-stimulated THP-1 were responsible for PCa migration. Importantly, TREM-1 signaling inhibitory peptide LP17 could block the R1881-stimulated production of these chemokines in THP-1 cells and attenuate the associated PCa migration.

## The Role of Androgen Receptor in Cross Talk Between Natural Killer Cells and Prostate Cancer

Natural killer (NK) cells are cytotoxic lymphocytes which play an important role in the regulation of innate immune response (Vivier et al., 2011; Perera Molligoda Arachchige, 2021). Accumulating evidence suggests that the infiltration of NK cells is greater in PCa than that in normal prostate tissues (Lin et al., 2017; Wu et al., 2020) and castration amplifies this phenomenon. By using the coculture system, Galustian et al. confirmed that PCa cells had a stronger ability to enhance IL-15-mediated expansion and cytotoxicity of NK cells than non-cancerous cell lines (PNT2 and WPMY-1) (Sakellariou et al., 2020), suggesting that the human body applies a protective strategy against PCa by activating NK cells. Accordantly, another study showed that AR could transcriptionally regulate the expression of NK inhibitory ligand LLT1 (lectin-like transcript 1) in PCa cells (Tang et al., 2020). Castration led to a short decline of AR activity so that the LLT1 level was reduced, eventually contributing to the expansion and activation of NK cells.

Specifically, the recruited NK cells could suppress the progression of CRPC by selectively degrading ARv7 (Lin et al., 2017), the most important AR variant determining CRPC growth and drug resistance (Antonarakis et al., 2014; Scher et al., 2016; Wang et al., 2017). NK cells could not only sensitize CRPC cells to anti-androgen treatment but also inhibit cell invasion of CRPC cells. More importantly, the suppression effect of NK cells on CRPC progression could be attenuated by the introduction of ARv7 into PCa cells. NK cells allowed CRPC cells to express high levels of miR-34 and miR-449, which bound the 3′-UTR of ARv7 and caused its degradation (Lin et al., 2017). As the direct downstream effect of ARv7, EZH2 was reduced upon NK cell treatment and was the key causal factor controlling PCa invasion. All these data indicate that targeting ARv7 or EZH2 may overcome CRPC progression.

## Cross Talk Between Mast Cells and Prostate Cancer

Mast cells are immune cells originated from myeloid stem cells. The wide distribution of mast cells across various tissues suggests that they act as key players to regulate a variety of physiological and pathological functions (Halova et al., 2012; Krystel-Whittemore et al., 2015). Typically, mast cells can be classified into intra-tumoral and peri-tumoral mast cells based on their location in specific tissues. Intra-tumoral mast cells exert a protective role to prevent cancer development while peri-tumoral mast cells support tumor growth (Fleischmann et al., 2009). Evidence supports the tumor-promoting role of mast cells in PCa owing to their peri-tumoral addiction (Johansson et al., 2010). Castration with ADT stimulates mast cell recruitment. Chang et al. used the coculture system to identify several chemoattractants including IL8, adrenomedullin (AM), and CCL8 as key molecules indispensable for mast cell recruitment (Dang et al., 2015). Interestingly, the expression levels of these cytokines were tightly regulated by AR. Castration-mediated AR inhibition increased the expression levels of IL8, AM, and CCL8 in PCa cells so that more mast cells were attracted. Their results also revealed that the recruited mast cells could drive neuroendocrine (NE) and stemness differentiation of PCa cells (Li et al., 2015), promoting cancer invasion and tumor metastasis. According to their data, miR-32 upregulation in PCa after AR inhibition was the causal factor determining NE differentiation. However, the targets of miR-32, potentially the direct downstream effectors regulating NE differentiation, are still not yet identified.

Evidence also revealed that infiltrating mast cells could enhance the occupation of the HOTAIR/PRC2-suppressing complex at the upstream promoter region of the AR gene locus, leading to the suppressive transcription of AR (Li et al., 2015). The exact role of AR in mast cells in regard to PCa growth and invasion/metastasis is another direction for further investigation.

## Conclusion and Future Perspective

**FIGURE 3:**
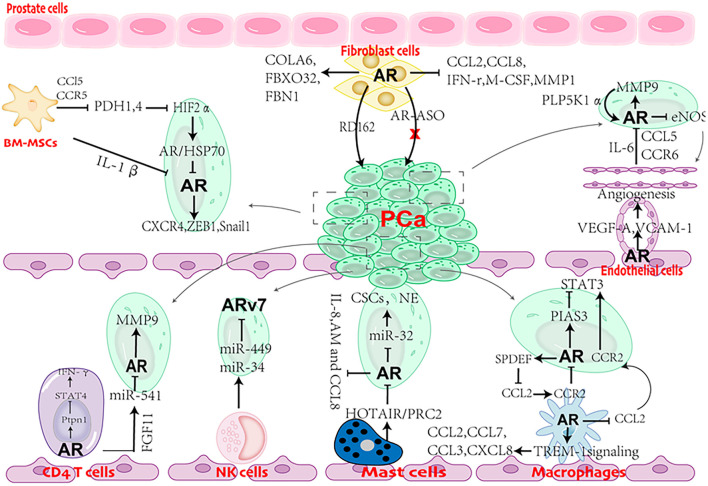
Cartoon showing the role of AR in cross talks between stromal cells and prostate cancer (PCa) cells.

Although clinical evidence suggests that stromal AR is gradually lost as PCa progresses to advanced stage, experimental results indicate that the role of AR in stromal cells is complicated (Table 2) and is still open for investigation. Stromal cells (CAFs, BM-MSCs, endothelial cells, CD4 + T cells, macrophages, NK cells, and mast cells) are clearly observed in PCa and castration with ADT or anti-androgen treatment further increasing the infiltration, leading to AR reduction (Figure 3). Although AR reduction retards PCa growth, it promotes PCa cell invasion and tumor metastasis. Further explorations support that AR inhibition increases the populations of cancer stem-like cells and neuroendocrine cells, which are widely viewed as AR negative and lethal type of PCa cells with little response to current treatments. Besides, AR inhibition also releases the transcriptional suppression on MMP9 and multiple cytokines (CCL5, CCL2 IL8, IL-1β and more). These cytokines serve as chemoattractants to recruit stromal cells into PCa. MMP9, as one of matrix metallopeptidases, can degrade the ECM and allow PCa cells to invade. From this point of view, although ADT can slow down PCa tumor growth, it predisposes PCa to undergo malignant transformation. Therefore, a simultaneous inhibition of AR signaling and the supporting stimuli from the surrounding microenvironment or signaling pathways involved in tumor metastasis is a promising therapeutic strategy to battle against PCa progression.

**TABLE 2:** The role of AR in PCa stromal cells.

Cell type	The role of AR	Effect on PCa	References
Endothelial cells	Is involved in TNF-induced apoptosis	Inhibits progression	Ling et al., 2002
	Induces VEGF-A and VCAM-1 expression, promotes proliferation	Promotes progression	Cai J. et al., 2011; Eisermann et al., 2013; Torres-Estay et al., 2017
CD4 T cells	Decreases IFN-γ production and suppresses CD4 T cell proliferation	Inhibits progression	Kissick et al., 2014
Fibroblasts	Upregulates FGF-2 and FGF-10 levels	Promotes initiation	Ricke et al., 2012
	Decreases cell proliferation while increase cell migration of fibroblasts cells		Castoria et al., 2011, 2014
	Negatively regulates the expression levels of CCL2, CCL8, M-CSF and IFN-γ	Inhibits invasion	Cioni et al., 2018; Palethorpe et al., 2018
	Regulates cytokines secretion	Increases growth invasion	Niu et al., 2008; Liao et al., 2017
Macrophages	Negatively regulates the expression levels of CCL2	Inhibits invasion	Izumi et al., 2013
	Promotes M2 macrophages polarization and regulates TREM-1 signaling	Promotes invasion	Becerra-Diaz et al., 2018; Cioni et al., 2020

## Author Contributions

QT and BC drafted the manuscript. RD and RW oversaw this manuscript. All authors contributed to the article and approved the submitted version.

## Conflict of Interest

The authors declare that the research was conducted in the absence of any commercial or financial relationships that could be construed as a potential conflict of interest.

## Publisher’s Note

All claims expressed in this article are solely those of the authors and do not necessarily represent those of their affiliated organizations, or those of the publisher, the editors and the reviewers. Any product that may be evaluated in this article, or claim that may be made by its manufacturer, is not guaranteed or endorsed by the publisher.

## References

[B1] Antonarakis E. S. Lu C. Wang H. Luber B. Nakazawa M. Roeser J. C. (2014). AR-V7 and resistance to enzalutamide and abiraterone in prostate cancer. *N. Engl. J. Med.* 371 1028–1038. 25184630 10.1056/NEJMoa1315815 PMC4201502

[B2] Ardiani A. Gameiro S. R. Kwilas A. R. Donahue R. N. Hodge J. W. (2014). Androgen deprivation therapy sensitizes prostate cancer cells to T-cell killing through androgen receptor dependent modulation of the apoptotic pathway. *Oncotarget* 5 9335–9348. 10.18632/oncotarget.2429 25344864 PMC4253438

[B3] Becerra-Diaz M. Strickland A. B. Keselman A. Heller N. M. (2018). Androgen and androgen receptor as enhancers of M2 macrophage polarization in allergic lung inflammation. *J. Immunol.* 201 2923–2933. 10.4049/jimmunol.1800352 30305328 PMC6219904

[B4] Brennen W. N. Zhang B. Kulac I. Kisteman L. N. Antony L. Wang H. (2017). Mesenchymal stem cell infiltration during neoplastic transformation of the human prostate. *Oncotarget* 8 46710–46727. 10.18632/oncotarget.17362 28493842 PMC5564518

[B5] Cai H. Babic I. Wei X. Huang J. Witte O. N. (2011). Invasive prostate carcinoma driven by c-Src and androgen receptor synergy. *Cancer Res.* 71 862–872. 10.1158/0008-5472.can-10-1605 21135112 PMC3032821

[B6] Cai J. Hong Y. Weng C. Tan C. Imperato-McGinley J. Zhu Y. S. (2011). Androgen stimulates endothelial cell proliferation via an androgen receptor/VEGF/cyclin A-mediated mechanism. *Am. J. Physiol. Heart Circ. Physiol.* 300 H1210–H1221. 21257919 10.1152/ajpheart.01210.2010 PMC3075033

[B7] Castoria G. Auricchio F. Migliaccio A. (2017). Extranuclear partners of androgen receptor: at the crossroads of proliferation, migration, and neuritogenesis. *FASEB J.* 31 1289–1300. 10.1096/fj.201601047r 28031322

[B8] Castoria G. D’Amato L. Ciociola A. Giovannelli P. Giraldi T. Sepe L. (2011). Androgen-induced cell migration: role of androgen receptor/filamin A association. *PLoS One* 6:e17218. 10.1371/journal.pone.0017218 21359179 PMC3040221

[B9] Castoria G. Giovannelli P. Di Donato M. Ciociola A. Hayashi R. Bernal F. (2014). Role of non-genomic androgen signalling in suppressing proliferation of fibroblasts and fibrosarcoma cells. *Cell Death Dis.* 5:e1548. 10.1038/cddis.2014.497 25476896 PMC4649827

[B10] Cha H. R. Lee J. H. Hensel J. A. Sawant A. B. Davis B. H. Lee C. M. (2016). Prostate cancer-derived cathelicidin-related antimicrobial peptide facilitates macrophage differentiation and polarization of immature myeloid progenitors to protumorigenic macrophages. *Prostate* 76 624–636. 10.1002/pros.23155 26856684 PMC5551898

[B11] Chang M. A. Patel V. Gwede M. Morgado M. Tomasevich K. Fong E. L. (2014). IL-1beta induces p62/SQSTM1 and represses androgen receptor expression in prostate cancer cells. *J. Cell Biochem.* 115 2188–2197. 25103771 10.1002/jcb.24897 PMC4517682

[B12] Chen T. Wang L. H. Farrar W. L. (2000). Interleukin 6 activates androgen receptor-mediated gene expression through a signal transducer and activator of transcription 3-dependent pathway in LNCaP prostate cancer cells. *Cancer Res.* 60 2132–2135. 10786674

[B13] Cioni B. Nevedomskaya E. Melis M. H. M. van Burgsteden J. Stelloo S. Hodel E. (2018). Loss of androgen receptor signaling in prostate cancer-associated fibroblasts (CAFs) promotes CCL2- and CXCL8-mediated cancer cell migration. *Mol. Oncol.* 12 1308–1323. 10.1002/1878-0261.12327 29808619 PMC6068356

[B14] Cioni B. Zaalberg A. van Beijnum J. R. Melis M. H. M. van Burgsteden J. Muraro M. J. (2020). Androgen receptor signalling in macrophages promotes TREM-1-mediated prostate cancer cell line migration and invasion. *Nat. Commun.* 11:4498. 10.1038/s41467-020-18313-y PMC7481219 32908142

[B15] Comito G. Giannoni E. Segura C. P. Barcellos-de-Souza P. Raspollini M. R. Baroni G. (2014). Cancer-associated fibroblasts and M2-polarized macrophages synergize during prostate carcinoma progression. *Oncogene* 33 2423–2431. 10.1038/onc.2013.191 23728338

[B16] Crawford E. D. Hou A. H. (2009). The role of LHRH antagonists in the treatment of prostate cancer. *Oncology* 23 626–630. 19626830

[B17] Dang Q. Li L. Xie H. He D. Chen J. Song W. (2015). Anti-androgen enzalutamide enhances prostate cancer neuroendocrine (NE) differentiation via altering the infiltrated mast cells –> androgen receptor (AR) –> miRNA32 signals. *Mol. Oncol.* 9 1241–1251. 10.1016/j.molonc.2015.02.010 25817444 PMC5528811

[B18] DePrimo S. E. Diehn M. Nelson J. B. Reiter R. E. Matese J. Fero M. (2002). Transcriptional programs activated by exposure of human prostate cancer cells to androgen. *Genome Biol.* 3:RESEARCH0032. 10.1186/gb-2002-3-7-research0032 PMC126237 12184806

[B19] Di Donato M. Zamagni A. Galasso G. Di Zazzo E. Giovannelli P. Barone M. V. (2021). The androgen receptor/filamin A complex as a target in prostate cancer microenvironment. *Cell Death Dis.* 12:127. 10.1038/s41419-021-03402-7 PMC7838283 33500395

[B20] Dudley A. C. (2012). Tumor endothelial cells. *Cold Spring Harb. Perspect. Med.* 2:a006536. 10.1101/cshperspect.a006536 PMC3282494 22393533

[B21] Eisermann K. Broderick C. J. Bazarov A. Moazam M. M. Fraizer G. C. (2013). Androgen up-regulates vascular endothelial growth factor expression in prostate cancer cells via an Sp1 binding site. *Mol. Cancer* 12:7. 10.1186/1476-4598-12-7 PMC3616929 23369005

[B22] Fang L. Y. Izumi K. Lai K. P. Liang L. Li L. Miyamoto H. (2013). Infiltrating macrophages promote prostate tumorigenesis via modulating androgen receptor-mediated CCL4-STAT3 signaling. *Cancer Res.* 73 5633–5646. 10.1158/0008-5472.can-12-3228 23878190 PMC3833080

[B23] Fleischmann A. Schlomm T. Kollermann J. Sekulic N. Huland H. Mirlacher M. (2009). Immunological microenvironment in prostate cancer: high mast cell densities are associated with favorable tumor characteristics and good prognosis. *Prostate* 69 976–981. 10.1002/pros.20948 19274666

[B24] Godoy A. Montecinos V. P. Gray D. R. Sotomayor P. Yau J. M. Vethanayagam R. R. (2011). Androgen deprivation induces rapid involution and recovery of human prostate vasculature. *Am. J. Physiol. Endocrinol. Metab.* 300 E263–E275. 20699437 10.1152/ajpendo.00210.2010 PMC3280699

[B25] Halova I. Draberova L. Draber P. (2012). Mast cell chemotaxis - chemoattractants and signaling pathways. *Front. Immunol.* 3:119. 10.3389/fimmu.2012.00119 22654878 PMC3360162

[B26] Heinlein C. A. Chang C. (2004). Androgen receptor in prostate cancer. *Endocr. Rev.* 25 276–308. 15082523 10.1210/er.2002-0032

[B27] Henshall S. M. Quinn D. I. Lee C. S. Head D. R. Golovsky D. Brenner P. C. (2001). Altered expression of androgen receptor in the malignant epithelium and adjacent stroma is associated with early relapse in prostate cancer. *Cancer Res.* 61 423–427. 11212224

[B28] Hu S. Li L. Yeh S. Cui Y. Li X. Chang H. C. (2015). Infiltrating T cells promote prostate cancer metastasis via modulation of FGF11–>miRNA-541–>androgen receptor (AR)–>MMP9 signaling. *Mol. Oncol.* 9 44–57. 10.1016/j.molonc.2014.07.013 25135278 PMC4277919

[B29] Izumi K. Fang L. Y. Mizokami A. Namiki M. Li L. Lin W. J. (2013). Targeting the androgen receptor with siRNA promotes prostate cancer metastasis through enhanced macrophage recruitment via CCL2/CCR2-induced STAT3 activation. *EMBO Mol. Med.* 5 1383–1401. 10.1002/emmm.201202367 23982944 PMC3799493

[B30] Johansson A. Rudolfsson S. Hammarsten P. Halin S. Pietras K. Jones J. (2010). Mast cells are novel independent prognostic markers in prostate cancer and represent a target for therapy. *Am. J. Pathol.* 177 1031–1041. 10.2353/ajpath.2010.100070 20616342 PMC2913352

[B31] Kissick H. T. Sanda M. G. Dunn L. K. Pellegrini K. L. On S. T. Noel J. K. (2014). Androgens alter T-cell immunity by inhibiting T-helper 1 differentiation. *Proc. Natl. Acad. Sci. U.S.A.* 111 9887–9892. 10.1073/pnas.1402468111 24958858 PMC4103356

[B32] Koivisto P. Kononen J. Palmberg C. Tammela T. Hyytinen E. Isola J. (1997). Androgen receptor gene amplification: a possible molecular mechanism for androgen deprivation therapy failure in prostate cancer. *Cancer Res.* 57 314–319. 9000575

[B33] Krystel-Whittemore M. Dileepan K. N. Wood J. G. (2015). Mast cell: a multi-functional master cell. *Front. Immunol.* 6:620. 10.3389/fimmu.2015.00620 26779180 PMC4701915

[B34] Larsson P. Syed Khaja A. S. Semenas J. Wang T. Sarwar M. Dizeyi N. (2020). The functional interlink between AR and MMP9/VEGF signaling axis is mediated through PIP5K1alpha/pAKT in prostate cancer. *Int. J. Cancer* 146 1686–1699. 10.1002/ijc.32607 31381135 PMC7004098

[B35] Leach D. A. Need E. F. Toivanen R. Trotta A. P. Palethorpe H. M. Tamblyn D. J. (2015). Stromal androgen receptor regulates the composition of the microenvironment to influence prostate cancer outcome. *Oncotarget* 6 16135–16150. 10.18632/oncotarget.3873 25965833 PMC4599261

[B36] Lepor H. Shore N. D. (2012). LHRH agonists for the treatment of prostate cancer: 2012. *Rev. Urol.* 14 1–12. 10.1016/j.eursup.2005.04.002 23172994 PMC3503273

[B37] Li L. Dang Q. Xie H. Yang Z. He D. Liang L. (2015). Infiltrating mast cells enhance prostate cancer invasion via altering LncRNA-HOTAIR/PRC2-androgen receptor (AR)-MMP9 signals and increased stem/progenitor cell population. *Oncotarget* 6 14179–14190. 10.18632/oncotarget.3651 25895025 PMC4546459

[B38] Li Y. Li C. X. Ye H. Chen F. Melamed J. Peng Y. (2008). Decrease in stromal androgen receptor associates with androgen-independent disease and promotes prostate cancer cell proliferation and invasion. *J. Cell Mol. Med.* 12 2790–2798. 10.1111/j.1582-4934.2008.00279.x 18266956 PMC3828892

[B39] Liao C. P. Chen L. Y. Luethy A. Kim Y. Kani K. MacLeod A. R. (2017). Androgen receptor in cancer-associated fibroblasts influences stemness in cancer cells. *Endocr. Relat. Cancer* 24 157–170. 10.1530/erc-16-0138 28264911 PMC5453797

[B40] Lin S. J. Chou F. J. Li L. Lin C. Y. Yeh S. Chang C. (2017). Natural killer cells suppress enzalutamide resistance and cell invasion in the castration resistant prostate cancer via targeting the androgen receptor splicing variant 7 (ARv7). *Cancer Lett.* 398 62–69. 10.1016/j.canlet.2017.03.035 28373004

[B41] Lin T. H. Izumi K. Lee S. O. Lin W. J. Yeh S. Chang C. (2013). Anti-androgen receptor ASC-J9 versus anti-androgens MDV3100 (Enzalutamide) or Casodex (Bicalutamide) leads to opposite effects on prostate cancer metastasis via differential modulation of macrophage infiltration and STAT3-CCL2 signaling. *Cell Death Dis.* 4:e764. 10.1038/cddis.2013.270 23928703 PMC3763432

[B42] Ling S. Dai A. Williams M. R. Myles K. Dilley R. J. Komesaroff P. A. (2002). Testosterone (T) enhances apoptosis-related damage in human vascular endothelial cells. *Endocrinology* 143 1119–1125. 10.1210/endo.143.3.8679 11861539

[B43] Linxweiler J. Hajili T. Korbel C. Berchem C. Zeuschner P. Muller A. (2020). Cancer-associated fibroblasts stimulate primary tumor growth and metastatic spread in an orthotopic prostate cancer xenograft model. *Sci. Rep.* 10:12575. 10.1038/s41598-020-69424-x PMC7387494 32724081

[B44] Liu Y. Karaca M. Zhang Z. Gioeli D. Earp H. S. Whang Y. E. (2010). Dasatinib inhibits site-specific tyrosine phosphorylation of androgen receptor by Ack1 and Src kinases. *Oncogene* 29 3208–3216. 10.1038/onc.2010.103 20383201 PMC2880659

[B45] Lonergan P. E. Tindall D. J. (2011). Androgen receptor signaling in prostate cancer development and progression. *J. Carcinog.* 10:20. 10.4103/1477-3163.83937 21886458 PMC3162670

[B46] Loy C. J. Sim K. S. Yong E. L. (2003). Filamin-A fragment localizes to the nucleus to regulate androgen receptor and coactivator functions. *Proc. Natl. Acad. Sci. U.S.A.* 100 4562–4567. 10.1073/pnas.0736237100 12682292 PMC153595

[B47] Luo J. Lee S. O. Cui Y. Yang R. Li L. Chang C. (2015). Infiltrating bone marrow mesenchymal stem cells (BM-MSCs) increase prostate cancer cell invasion via altering the CCL5/HIF2alpha/androgen receptor signals. *Oncotarget* 6 27555–27565. 26342197 10.18632/oncotarget.4515 PMC4695008

[B48] Luo J. Ok Lee S. Liang L. Huang C. K. Li L. Wen S. (2014). Infiltrating bone marrow mesenchymal stem cells increase prostate cancer stem cell population and metastatic ability via secreting cytokines to suppress androgen receptor signaling. *Oncogene* 33 2768–2778. 10.1038/onc.2013.233 23792449

[B49] McArdle P. A. Canna K. McMillan D. C. McNicol A. M. Campbell R. Underwood M. A. (2004). The relationship between T-lymphocyte subset infiltration and survival in patients with prostate cancer. *Br. J. Cancer* 91 541–543. 10.1038/sj.bjc.6601943 15266325 PMC2409839

[B50] Mendez-Ferrer S. Michurina T. V. Ferraro F. Mazloom A. R. Macarthur B. D. Lira S. A. (2010). Mesenchymal and haematopoietic stem cells form a unique bone marrow niche. *Nature* 466 829–834. 10.1038/nature09262 20703299 PMC3146551

[B51] Mercader M. Bodner B. K. Moser M. T. Kwon P. S. Park E. S. Manecke R. G. (2001). T cell infiltration of the prostate induced by androgen withdrawal in patients with prostate cancer. *Proc. Natl. Acad. Sci. U.S.A.* 98 14565–14570. 11734652 10.1073/pnas.251140998 PMC64722

[B52] Migliaccio A. Castoria G. Di Domenico M. de Falco A. Bilancio A. Lombardi M. (2000). Steroid-induced androgen receptor-oestradiol receptor beta-Src complex triggers prostate cancer cell proliferation. *EMBO J.* 19 5406–5417. 10.1093/emboj/19.20.5406 11032808 PMC314017

[B53] Migliaccio A. Di Domenico M. Castoria G. Nanayakkara M. Lombardi M. de Falco A. (2005). Steroid receptor regulation of epidermal growth factor signaling through Src in breast and prostate cancer cells: steroid antagonist action. *Cancer Res.* 65 10585–10593. 10.1158/0008-5472.can-05-0912 16288052

[B54] Montgomery R. B. Mostaghel E. A. Vessella R. Hess D. L. Kalhorn T. F. Higano C. S. (2008). Maintenance of intratumoral androgens in metastatic prostate cancer: a mechanism for castration-resistant tumor growth. *Cancer Res.* 68 4447–4454. 10.1158/0008-5472.can-08-0249 18519708 PMC2536685

[B55] Nakazawa M. Antonarakis E. S. Luo J. (2014). Androgen receptor splice variants in the era of enzalutamide and abiraterone. *Horm. Cancer* 5 265–273. 10.1007/s12672-014-0190-1 25048254 PMC4167475

[B56] Niu Y. Altuwaijri S. Lai K. P. Wu C. T. Ricke W. A. Messing E. M. (2008). Androgen receptor is a tumor suppressor and proliferator in prostate cancer. *Proc. Natl. Acad. Sci. U.S.A.* 105 12182–12187. 18723679 10.1073/pnas.0804700105 PMC2527886

[B57] Olapade-Olaopa E. O. MacKay E. H. Taub N. A. Sandhu D. P. Terry T. R. Habib F. K. (1999). Malignant transformation of human prostatic epithelium is associated with the loss of androgen receptor immunoreactivity in the surrounding stroma. *Clin. Cancer Res.* 5 569–576. 10100708

[B58] Palethorpe H. M. Leach D. A. Need E. F. Drew P. A. Smith E. (2018). Myofibroblast androgen receptor expression determines cell survival in co-cultures of myofibroblasts and prostate cancer cells in vitro. *Oncotarget* 9 19100–19114. 10.18632/oncotarget.24913 29721186 PMC5922380

[B59] Perera Molligoda Arachchige A. S. (2021). Human NK cells: from development to effector functions. *Innate Immun.* 27 212–229. 10.1177/17534259211001512 33761782 PMC8054151

[B60] Peterziel H. Mink S. Schonert A. Becker M. Klocker H. Cato A. C. (1999). Rapid signalling by androgen receptor in prostate cancer cells. *Oncogene* 18 6322–6329. 10.1038/sj.onc.1203032 10597231

[B61] Pidsley R. Lawrence M. G. Zotenko E. Niranjan B. Statham A. Song J. (2018). Enduring epigenetic landmarks define the cancer microenvironment. *Genome Res.* 28 625–638. 10.1101/gr.229070.117 29650553 PMC5932604

[B62] Pittenger M. F. Mackay A. M. Beck S. C. Jaiswal R. K. Douglas R. Mosca J. D. (1999). Multilineage potential of adult human mesenchymal stem cells. *Science* 284 143–147. 10102814 10.1126/science.284.5411.143

[B63] Placencio V. R. Li X. Sherrill T. P. Fritz G. Bhowmick N. A. (2010). Bone marrow derived mesenchymal stem cells incorporate into the prostate during regrowth. *PLoS One* 5:e12920. 10.1371/journal.pone.0012920 20886110 PMC2944821

[B64] Ricciardelli C. Choong C. S. Buchanan G. Vivekanandan S. Neufing P. Stahl J. (2005). Androgen receptor levels in prostate cancer epithelial and peritumoral stromal cells identify non-organ confined disease. *Prostate* 63 19–28. 10.1002/pros.20154 15378523

[B65] Ricke E. A. Williams K. Lee Y. F. Couto S. Wang Y. Hayward S. W. (2012). Androgen hormone action in prostatic carcinogenesis: stromal androgen receptors mediate prostate cancer progression, malignant transformation and metastasis. *Carcinogenesis* 33 1391–1398. 10.1093/carcin/bgs153 22535887 PMC3499049

[B66] Roumiguie M. Paoletti X. Neuzillet Y. Mathieu R. Vincendeau S. Kleinclauss F. (2021). Apalutamide, darolutamide and enzalutamide in nonmetastatic castration-resistant prostate cancer: a meta-analysis. *Future Oncol.* 17 1811–1823. 10.2217/fon-2020-1104 33543650

[B67] Ruizeveld de Winter J. A. Janssen P. J. Sleddens H. M. Verleun-Mooijman M. C. Trapman J. Brinkmann A. O. (1994). Androgen receptor status in localized and locally progressive hormone refractory human prostate cancer. *Am. J. Pathol.* 144 735–746. 7512791 PMC1887232

[B68] Sahai E. Astsaturov I. Cukierman E. DeNardo D. G. Egeblad M. Evans R. M. (2020). A framework for advancing our understanding of cancer-associated fibroblasts. *Nat. Rev. Cancer* 20 174–186. 10.1038/s41568-019-0238-1 31980749 PMC7046529

[B69] Sakellariou C. Elhage O. Papaevangelou E. Giustarini G. Esteves A. M. Smolarek D. (2020). Prostate cancer cells enhance interleukin-15-mediated expansion of NK cells. *BJU Int.* 125 89–102. 10.1111/bju.14893 31392791

[B70] Sanchez C. Chan R. Bajgain P. Rambally S. Palapattu G. Mims M. (2013). Combining T-cell immunotherapy and anti-androgen therapy for prostate cancer. *Prostate Cancer Prostatic Dis.* 16 123–31,S1. 23295316 10.1038/pcan.2012.49 PMC3883310

[B71] Scher H. I. Beer T. M. Higano C. S. Anand A. Taplin M. E. Efstathiou E. (2010). Antitumour activity of MDV3100 in castration-resistant prostate cancer: a phase 1-2 study. *Lancet* 375 1437–1446. 20398925 10.1016/S0140-6736(10)60172-9 PMC2948179

[B72] Scher H. I. Buchanan G. Gerald W. Butler L. M. Tilley W. D. (2004). Targeting the androgen receptor: improving outcomes for castration-resistant prostate cancer. *Endocr. Relat. Cancer* 11 459–476. 10.1677/erc.1.00525 15369448

[B73] Scher H. I. Lu D. Schreiber N. A. Louw J. Graf R. P. Vargas H. A. (2016). Association of AR-V7 on circulating tumor cells as a treatment-specific biomarker with outcomes and survival in castration-resistant prostate cancer. *JAMA Oncol.* 2 1441–1449. 10.1001/jamaoncol.2016.1828 27262168 PMC5206761

[B74] Schweizer M. T. Antonarakis E. S. (2012). Abiraterone and other novel androgen-directed strategies for the treatment of prostate cancer: a new era of hormonal therapies is born. *Ther. Adv. Urol.* 4 167–178. 10.1177/1756287212452196 22852027 PMC3398601

[B75] Seaton A. Scullin P. Maxwell P. J. Wilson C. Pettigrew J. Gallagher R. (2008). Interleukin-8 signaling promotes androgen-independent proliferation of prostate cancer cells via induction of androgen receptor expression and activation. *Carcinogenesis* 29 1148–1156. 10.1093/carcin/bgn109 18487223

[B76] Sejda A. Sigorski D. Gulczynski J. Wesolowski W. Kitlinska J. Izycka-Swieszewska E. (2020). Complexity of neural component of tumor microenvironment in prostate cancer. *Pathobiology* 87 87–99. 10.1159/000505437 32045912

[B77] Shabsigh A. Chang D. T. Heitjan D. F. Kiss A. Olsson C. A. Puchner P. J. (1998). Rapid reduction in blood flow to the rat ventral prostate gland after castration: preliminary evidence that androgens influence prostate size by regulating blood flow to the prostate gland and prostatic endothelial cell survival. *Prostate* 36 201–206. 10.1002/(sici)1097-0045(19980801)36:3<201::aid-pros9>3.0.co;2-j 9687993

[B78] Sica A. Larghi P. Mancino A. Rubino L. Porta C. Totaro M. G. (2008). Macrophage polarization in tumour progression. *Semin. Cancer Biol.* 18 349–355. 18467122 10.1016/j.semcancer.2008.03.004

[B79] Siegel R. L. Miller K. D. Jemal A. (2020). Cancer statistics, 2020. *CA Cancer J. Clin.* 70 7–30. 31912902 10.3322/caac.21590

[B80] Smith D. F. Toft D. O. (2008). Minireview: the intersection of steroid receptors with molecular chaperones: observations and questions. *Mol. Endocrinol.* 22 2229–2240. 10.1210/me.2008-0089 18451092 PMC2582531

[B81] Tan M. H. Li J. Xu H. E. Melcher K. Yong E. L. (2015). Androgen receptor: structure, role in prostate cancer and drug discovery. *Acta Pharmacol. Sin.* 36 3–23. 10.1038/aps.2014.18 24909511 PMC4571323

[B82] Tang M. Gao S. Zhang L. Liu B. Li J. Wang Z. (2020). Docetaxel suppresses immunotherapy efficacy of natural killer cells toward castration-resistant prostate cancer cells via altering androgen receptor-lectin-like transcript 1 signals. *Prostate* 80 742–752. 10.1002/pros.23988 32449811

[B83] Taylor R. A. Toivanen R. Frydenberg M. Pedersen J. Harewood L. Australian Prostate Cancer Bioresource. (2012). Human epithelial basal cells are cells of origin of prostate cancer, independent of CD133 status. *Stem Cells* 30 1087–1096. 10.1002/stem.1094 22593016

[B84] Thienger P. Rubin M. A. (2021). Prostate cancer hijacks the microenvironment. *Nat. Cell Biol.* 23 3–5. 10.1038/s41556-020-00616-3 33420486

[B85] Tilley W. D. Buchanan G. Hickey T. E. Bentel J. M. (1996). Mutations in the androgen receptor gene are associated with progression of human prostate cancer to androgen independence. *Clin. Cancer Res.* 2 277–285. 9816170

[B86] Tomic T. T. Gustavsson H. Wang W. Jennbacken K. Welen K. Damber J. E. (2012). Castration resistant prostate cancer is associated with increased blood vessel stabilization and elevated levels of VEGF and Ang-2. *Prostate* 72 705–712. 10.1002/pros.21472 21809353

[B87] Torres-Estay V. Carreno D. V. Fuenzalida P. Watts A. San Francisco I. F. Montecinos V. P. (2017). Androgens modulate male-derived endothelial cell homeostasis using androgen receptor-dependent and receptor-independent mechanisms. *Angiogenesis* 20 25–38. 10.1007/s10456-016-9525-6 27679502

[B88] Tsai Y. C. Chen W. Y. Abou-Kheir W. Zeng T. Yin J. J. Bahmad H. (2018). Androgen deprivation therapy-induced epithelial-mesenchymal transition of prostate cancer through downregulating SPDEF and activating CCL2. *Biochim. Biophys. Acta Mol. Basis Dis.* 1864 1717–1727. 10.1016/j.bbadis.2018.02.016 29477409

[B89] Visakorpi T. Hyytinen E. Koivisto P. Tanner M. Keinanen R. Palmberg C. (1995). In vivo amplification of the androgen receptor gene and progression of human prostate cancer. *Nat. Genet.* 9 401–406. 10.1038/ng0495-401 7795646

[B90] Vivier E. Raulet D. H. Moretta A. Caligiuri M. A. Zitvogel L. Lanier L. L. (2011). Innate or adaptive immunity? The example of natural killer cells. *Science* 331 44–49. 21212348 10.1126/science.1198687 PMC3089969

[B91] Waghray A. Feroze F. Schober M. S. Yao F. Wood C. Puravs E. (2001). Identification of androgen-regulated genes in the prostate cancer cell line LNCaP by serial analysis of gene expression and proteomic analysis. *Proteomics* 1 1327–1338. 10.1002/1615-9861(200110)1:10<1327::aid-prot1327>3.0.co;2-b 11721644

[B92] Wang R. Sun Y. Li L. Niu Y. Lin W. Lin C. (2017). Preclinical study using malat1 small interfering RNA or androgen receptor splicing variant 7 degradation enhancer ASC-J9((R)) to suppress enzalutamide-resistant prostate cancer progression. *Eur. Urol.* 72 835–844. 10.1016/j.eururo.2017.04.005 28528814 PMC5802348

[B93] Wang X. Lee S. O. Xia S. Jiang Q. Luo J. Li L. (2013). Endothelial cells enhance prostate cancer metastasis via IL-6–>androgen receptor–>TGF-beta–>MMP-9 signals. *Mol. Cancer Ther.* 12 1026–1037. 10.1158/1535-7163.mct-12-0895 23536722 PMC3782851

[B94] Wikstrom P. Marusic J. Stattin P. Bergh A. (2009). Low stroma androgen receptor level in normal and tumor prostate tissue is related to poor outcome in prostate cancer patients. *Prostate* 69 799–809. 10.1002/pros.20927 19189305

[B95] Wu J. D. Haugk K. Woodke L. Nelson P. Coleman I. Plymate S. R. (2006). Interaction of IGF signaling and the androgen receptor in prostate cancer progression. *J. Cell Biochem.* 99 392–401. 16639715 10.1002/jcb.20929

[B96] Wu Z. Chen H. Luo W. Zhang H. Li G. Zeng F. (2020). The landscape of immune cells infiltrating in prostate Cancer. *Front. Oncol.* 10:517637. 10.3389/fonc.2020.517637I PMC7658630 33194581

[B97] Yu S. Jia L. Zhang Y. Wu D. Xu Z. Ng C. F. (2013). Increased expression of activated endothelial nitric oxide synthase contributes to antiandrogen resistance in prostate cancer cells by suppressing androgen receptor transactivation. *Cancer Lett.* 328 83–94. 10.1016/j.canlet.2012.09.006 22995070

[B98] Yu S. Jiang Y. Wan F. Wu J. Gao Z. Liu D. (2017). Immortalized cancer-associated fibroblasts promote prostate cancer carcinogenesis. Proliferation and Invasion. *Anticancer Res.* 37 4311–4318. 28739723 10.21873/anticanres.11824

[B99] Yuri P. Shigemura K. Kitagawa K. Hadibrata E. Risan M. Zulfiqqar A. (2020). Increased tumor-associated macrophages in the prostate cancer microenvironment predicted patients’ survival and responses to androgen deprivation therapies in Indonesian patients cohort. *Prostate Int.* 8 62–69. 10.1016/j.prnil.2019.12.001 32647642 PMC7335973

[B100] Zhao R. Bei X. Yang B. Wang X. Jiang C. Shi F. (2018). Endothelial cells promote metastasis of prostate cancer by enhancing autophagy. *J. Exp. Clin. Cancer Res.* 37:221. 10.1186/s13046-018-0884-2 PMC6131784 30200999

[B101] Zhu P. Baek S. H. Bourk E. M. Ohgi K. A. Garcia-Bassets I. Sanjo H. (2006). Macrophage/cancer cell interactions mediate hormone resistance by a nuclear receptor derepression pathway. *Cell* 124 615–629. 10.1016/j.cell.2005.12.032 16469706

